# Supplementary material for: The Prognostic Role of Human Papillomavirus and p16 Status in Penile Squamous Cell Carcinoma—A Systematic Review
Source: Cancers (Basel). 2023 Jul 21;15(14):3713. doi: 10.3390/cancers15143713 (PMC10378259; doi:10.3390/cancers15143713)
Supplement: Supplementary file 1 [file cancers-15-03713-s001.zip › SR_Supplemental Table S1.pdf]

Table S1. Supplement—New Castle-Ottawa Scoring for Studies Meeting Search Inclusion Criteria

Study	Subject Selection (Max 4)	Study Comparability (Max 2)	Assessment of Outcomes (Max 3)	Total Score
Wiener et al., 1992 [18]	****	**	*	7
(Artur) Bezerra et al., 2001 [15]	****	**	***	9
Lont et al., 2006 [19]	****	**	**	8
Guerrero et al., 2008 [20]	****	**	***	9
Scheiner et al., 2008 [21]	****	**	*	7
Ferrandiz-Pulido et al., 2013 [22]	****	**	*	7
Gunia et al., 2012 [23]	****	**	**	8
Bethune et al., 2012 [24]	****	**	***	9
Dilorenzo et al., 2013 [25]	****	**	**	8
de Fonseca et al., 2013 [26]	****	**	*	7
Hernandez et al, 2014 [27]	****	**	*	7
(Stephania) Bezerra et al., 2015 [28]	****	**	*	7
Djajadiningrat et al., 2015 [29]	****	**	**	8
McDaniel et al., 2015 [30]	****	**	*	7
Steinestel et al., 2015 [31]	****	**	*	7
Tang et al., 2015 [32]	****	**	*	7
Zargar-Shoshtari et al., 2016 [33]	****	**	*	7
Afonso et al., 2017 [34]	****	**	*	7
de Arau ̂jo et al., 2018 [35]	****	**	*	7
Ottenhof et al., 2018 [36]	****	**	***	9
Vicenilma Martins et al., 2018 [13]	****	**	**	8
Takamoto et al., 2018 [37]	****	**	***	9
De Bacco et al., 2020 [38]	****	**	***	9
Wang et al., 2020 [39]	***	*	**	6
Ashley et al., 2020 [40]	**	*	**	5
Pereira-Lourenço et al., 2020 [41]	****	**	**	8
Valqui ́ria Martins et al., 2020 [42]	****	**	**	8
Muresu et al., 2020 [43]	****	**	*	7
Chu et al., 2020 [44]	****	**	**	8
Chipollini et al., 2021 [45]	***	**	**	7
Mohanty et al., 2021 [46]	****	**	***	9
Müller et al., 2021 [47]	****	**	*	7
Browne et al., 2022 [48]	****	**	*	7
Chahoud et al., 2022 [4]	****	**	***	9
				**MEDIAN SCORE: 7**
