# Supplementary material for: The Prognostic Role of Human Papillomavirus and p16 Status in Penile Squamous Cell Carcinoma—A Systematic Review
Source: Cancers (Basel). 2023 Jul 21;15(14):3713. doi: 10.3390/cancers15143713 (PMC10378259; doi:10.3390/cancers15143713)
Supplement: Supplementary file 1 [file cancers-15-03713-s001.zip › SR_Supplemental Table S2.pdf]

Study Author & Date	Study Method	Country	# Of Centers	Date of Data Collection	Median Follow-up Duration	Total Sample Size (n)	Mean Age	p16 Detection Method	Type Of Tissue Collected	HPV Detection Method
Wiener et al., 1992 [18]	Retrospective Study	USA	Single Center	1970-1989	N/A	29	61	N/A	Fixed	PCR
(Artur) Bezerra et al., 2001 [15]	Retrospective Study	Brazil	Single Center	1953-1993	Not Given	82	52	N/A	Fixed	PCR
Lont et al., 2006 [19]	Retrospective Study	Netherlands	Single Center	1963-2001	95 Months	171	64	N/A	Fixed	PCR
Guerrero et al., 2008 [20]	Retrospective Study	Spain	Multicenter	1988-2007	72 Months	24	67.6	Unmethylated	Fixed	PCR
Scheiner et al., 2008 [21]	Prospective Study	Brazil	Multicenter	1995-2000	15 Months	80	57.6	N/A	Frozen	PCR
Ferrandiz-Pulido et al., 2013 [22]	Retrospective Study	Spain	Multicenter	1987-2010	29.4 Months	82	67.25	H -score => 50 %	Fixed	PCR
Gunia et al., 2012 [23]	Retrospective Study	Germany	Multicenter	1993-2010	32 Months	92	67.2	Staining score =>/1 (any p16 expression in tumor cells) Staining pattern 0 represented p16INK4a - tumors while staining patterns 1 to 3 represented p16INK4a + tumors. 0 – Absent p16INK4a expression in all epithelial cells, Pattern 1– Spotty, patchy, and discontinuous immunostaining in suprabasal epithelial cells; Pattern 2 – Comparatively more extensive but discontinuous suprabasal expression; Pattern 3 – Full-thickness, continuous immunostaining	Fixed	N/A
Bethune et al., 2012 [24]	Retrospective Study	Canada	Single Center	1997-2009	3.9Y ± 3.3Y (Mean)	43	63	The following parameters were used for the interpretation of p16INK4a: +, strong cytoplasmic and nuclear staining in > 30% of tumor cells; equivocal, strong staining in <30%	Paraffin-Embedded Blocks	N/A

								of tumor cells or weak to moderate staining in 30% of tumor cells		
Dilorenzo et al., 2013 [25]	Retrospective Study	Italy	Multicenter	2000-2011	24 Months	30	59.25	N/A	Fixed	ISH
de Fonseca et al., 2013 [26]	Prospective Study	Brazil	Single Center	2001-2008	20 Months	82	57.25	N/A	Fixed	PCR
Hernandez et al, 2014 [27]	Retrospective Study	USA	Multicenter	1998–2005	Not Given	79	Mean HPV-ve 72.7 SD: 15.3	N/A	Formalin-fixed paraffin-embedded (FFPE)	PCR
(Stephania) Bezerra et al., 2015 [28]	Retrospective Study	USA	Single Center	1985-2013	Not Given	53	65.26	Strong and diffuse nuclear and cytoplasmic p16 positivity in most tumor cells	Fixed	ISH
Djajadiningrat et al., 2015 [29]	Retrospective Study	Netherlands	Single Center	2001-2009	5.2 Years	212	62.75	N/A	Formalin-Fixed Paraffin-Embedded (FFPE)	PCR
McDaniel et al., 2015 [30]	Retrospective Study	USA	Single Center	2005-2013	15.36 Months	43	63	Moderate or strong staining intensity in the cytoplasm and nucleus	Fixed	PCR
Steinestel et al., 2015 [31]	Retrospective Study	Germany	Multicenter	1995-2012	15 Months	58	64.5	Intense confluent or focally scattered nuclear and/or cytoplasmic staining pattern	Fixed	PCR
Tang et al., 2015 [32]	Retrospective Study	USA	Single Center	1998-2013	30 Months	119	62.25	Diffuse, continuous, and strong nucleus and cytoplasmic staining of the neoplastic cells. Discontinuous, focal, and weak staining as well as the absence of staining were interpreted as - for P16	Fixed	N/A
Zargar-Shoshtari et al., 2016 [33]	Retrospective Study	USA	Single Center	1999-2013	22 Months	57	62	Significant p53 expression was defined as expression of moderate intensity at 50% or greater or any strong p53 expression (H score of >= 150)	N/A	ISH
Afonso et al., 2017 [34]	Cross-Sectional Study	Brazil	Multicenter	2005-2015	20 Months	122	58.5	Unmethylated	N/A	PCR
de Arau´jo et al., 2018 [35]	Retrospective Study	Brazil	Single Center	2003-2015	N/A	183	N/A	N/A	Paraffin Embedded Tumor Fragments	PCR
Ottenhof et al., 2018 [36]	Retrospective Study	Netherlands	Single Center	2001 - 2009	100.7 Months	213	65.8	N/A	Formalin-Fixed Paraffin-Embedded (FFPE)	PCR
Vicenilma Martins et al., 2018 [13]	Retrospective Study	Brazil	Multicenter	2014-2016	17.3 Months	55	61	4 p16INK4a expression patterns were found and were categorized as	Fixed	PCR

								follows: pattern 0, complete absence of staining in all neoplastic cells.<br>Pattern 1, irregular and discontinuous individual staining in some of the neoplastic cells;<br>Pattern 2, a more extensive, although discontinuous, staining pattern with small clusters of + neoplastic cells;<br>Pattern 3, continuous and complete cytoplasmic and nuclear staining in all neoplastic cells.<br>Only pattern 3 was considered + for p16INK4a expression		
Takamoto et al., 2018 [37]	Retrospective Study	Japan	Single Center	1990-2010	23.5 Months	44	68.6	N/A	N/A	ISH
De Bacco et al., 2020 [38]	Retrospective Study	Brazil	Single Center	1998 - 2016	46.87 Months	40	64.6	Expression for p16 was defined as - if positivity was lower than 10%.	Formalin-Fixed Paraffin-Embedded (FFPE)	NA
Wang et al., 2020 [39]	Retrospective Study	China	Multicenter	1998-2015	Median 48.9 Months	292	Not Given	N/A	Frozen	Not Given
Ashley et al., 2020 [40]	Retrospective Study	United Kingdom	Single Center	2008-2018	17.5 Months	137	61.75	The histopathological reports of the first biopsy when PeIN was diagnosed were used to determine p16 overexpression (p16+ or p16−).	N/A	Not Given
Pereira-Lourenço et al., 2020 [41]	Retrospective Study	Portugal	Single Center	2010-2020	63 Months	35	65.25	The classification of Cubila et al. (26) was adapted, and overexpression of p16INK4a was defined as diffuse, continuous, and strong nuclear and cytoplasmic staining of the neoplastic cells. Discontinuous, focal, and weak staining as well the absence of staining was interpreted as - for p16INK4a overexpression	The material was fixed in 10% formol, embedded in paraffin and stained with haematoxylin-eosin	NA
Valquı´ria Martins et al., 2020 [42]	Retrospective Study	Brazil	Single Center	2013-2018	35.4 Months	47	57.4	Positivity for p16INK4a was defined as unequivocally nuclear and cytoplasmic staining of at least 70% of the tumor cells	At the moment of surgery, 3-5mm^3 of tissue fragments (mass of 50-150mg) from the tumor was collected and stored	PCR

									in a dry plastic microtube free of DNAse and RNAse	
Muresu et al., 2020 [43]	Retrospective Study	Italy	Single Center	2002 - 2019	Not Given	32	68	Not given	Formalin-Fixed Paraffin-Embedded (FFPE) Specimens	PCR
Chu et al., 2020 [44]	Retrospective Study	China	Single Center	1999-2013	57 Months	226	53.5	The expression of P16INK4a was classified as four patterns: 0, no stain; 1, weak and individual; 2, moderate with small clusters; and 3, strong and diffuse (Fig. 1). Only pattern 3 was represented + for p16INK4a expression	Formalin-Fixed Paraffin-Embedded (FFPE)	PCR
Chipollini et al., 2021 [45]	Retrospective Study	USA	Multicenter	2010 - 2016	29.9 Months	825	63.75	N/A	N/A	Not Given
Mohanty et al., 2021 [46]	Retrospective Study	India	Not clear	2009-2017	44 Months	123	62.25	Overexpression of p16 ink4a was defined as a continuous strong nuclear and/or nuclear and cytoplasmic staining of > 50% of the tumor cells	Paraffin Tissue Block	ISH
Müller et al., 2021 [47]	Retrospective Study	Germany	Single Center	Not Given	Not Given	60	N/A	Block like positivity was considered +, whereas mosaic like pattern was considered -	Paraffin Sections	Quantus Fluorometer
Browne et al., 2022 [48]	Retrospective Study	Ireland	Single Center	2015-2020	17.5 Months	81	64	N/A	N/A	Not Given
Chahoud et al., 2022 [4]	Retrospective Study	USA	Single Center	1991-2017	69.6 Months	143	58.75	Only staining pattern 2 with > 75% staining and staining pattern 3 with > 75% staining were considered p16+	Formalin-Fixed Paraffin-Embedded (FFPE) Tissue	ISH
